# Human oligodendroglial cells express low levels of C1 inhibitor and membrane cofactor protein mRNAs

**DOI:** 10.1186/1742-2094-1-17

**Published:** 2004-08-24

**Authors:** Masato Hosokawa, Andis Klegeris, Patrick L McGeer

**Affiliations:** 1 Kinsmen Laboratory of Neurological Research, University of British Columbia, 2255 Wesbrook Mall, Vancouver, BC, V6T 1Z3, Canada

## Abstract

**Background:**

Oligodendrocytes, neurons, astrocytes, microglia, and endothelial cells are capable of synthesizing complement inhibitor proteins. Oligodendrocytes are vulnerable to complement attack, which is particularly observed in multiple sclerosis. This vulnerability may be related to a deficiency in their ability to express complement regulatory proteins.

**Methods:**

This study compared the expression level of complement inhibitor mRNAs by human oligodendrocytes, astrocytes and microglia using semi-quantitative RT-PCR.

**Results:**

Semi-quantitative RT-PCR analysis showed that C1 inhibitor (C1-inh) mRNA expression was dramatically lower in oligodendroglial cells compared with astrocytes and microglia. The mRNA expression level of membrane cofactor protein (MCP) by oligodendrocytes was also significantly lower than for other cell types.

**Conclusion:**

The lower mRNA expression of C1-inh and MCP by oligodendrocytes could contribute to their vulnerability in several neurodegenerative and inflammatory diseases of the central nervous system.

## Background

Resident brain cells including oligodendrocytes [1,2], astrocytes, astrocytomas, microglia, glioblastomas [3-14], neurons [15,16], neuroblastomas [17,18] and endothelial cells [19,20] express mRNAs for complement proteins. Although the role of complement expression by these cells remains unclear, local complement activation in the central nervous system (CNS) might damage these cells and contribute to the pathology in several inflammatory and neurodegenerative diseases including multiple sclerosis, Alzheimer's disease and progressive supranuclear palsy.

For self-protection, resident brain cells also express complement inhibitors, such as membrane cofactor protein (MCP), decay-accelerating factor (DAF), CD59, and C1-esterase inhibitor (C1-inh). The human HOG oligodendroglial cell line produces MCP, DAF, CD59, C1-inh and S-protein, but not complement receptor 1 (CR1) [1]. Human oligodendrocytes have been reported to express CD59 [21] and DAF, but not MCP, CR1, homologous restriction factor (HRF: C8 bp) or clusterin [22]. Astrocytes [23], neurons and Schwann cells have been reported to express CD59 [24] and neuroblastoma cell lines C1-inh [18]. Astrocytoma cell lines have been reported to express MCP, DAF, and CD59 [25,26].

In this study, the expression level of mRNAs for various complement inhibitors by human oligodendrocytes, astrocytes and microglia were compared by semi-quantitative PCR. We show that oligodendrocytes express extremely low levels of mRNA for C1-inh and significantly lower levels of mRNA for MCP compared with astrocytes and microglia. The expression level of mRNAs for CD59 and DAF showed no significant differences between the three cell types.

## Methods

### Cell culture: microglial- and astrocyte-enriched cultures

Human microglial and astrocytic cells were isolated from surgically resected temporal lobe tissues. We thank Dr. J. Maguire, Department of Pathology and Laboratory Medicine, Vancouver General Hospital for providing the surgical specimens. Isolation protocols described by De Groot *et al*. [27,28] were used with minor modifications. Tissues were placed in a sterile Petri dish, rinsed with Hank's balanced salt solution, and visible blood vessels were removed. After washing tissues two more times with Hank's balanced salt solution, tissues were chopped into small (<2 mm^3^) pieces with a sterile scalpel. The fragments were transferred into a 50 ml centrifuge tube containing 10 ml of 0.25% trypsin solution (Gibco-BRL, Life Technologies, Burlington, ON, Canada), and incubated at 37°C for 20 min. Subsequently DNase I (from bovine pancreas, Pharmacia Biotech, Baie d'Urfé, PQ, Canada) was added to reach a final concentration of 50 μg/ml. Tissues were incubated for an additional 10 min at 37°C. The cell suspension was diluted with 10 ml of Dulbecco's modified Eagle's medium (DMEM) and nutrient mixture F12 ham (DMEM-F12; Sigma-Aldrich, Oakville, ON, Canada) with 10% fetal bovine serum (FBS; Gibco-BRL, Life Technologies), and gently triturated by using a 10 ml pipette with a wide mouth. After centrifugation at 275 × g for 10 min, the cell pellet was resuspended in serum containing medium, triturated several times, and passed through a 100 μm nylon cell strainer (Becton Dickinson, Franklin Lakes, NJ). The cell suspension was then centrifuged once more (275 × g for 10 min), resuspended into 10 ml of DMEM-F12 with 10% FBS containing gentamicin (50 μg/ml, from Sigma), and plated onto uncoated 10 cm tissue culture plates (Becton Dickinson). Plates were placed in a humidified 5% CO_2_, 95% air atmosphere at 37°C for 2 hr in order to achieve adherence of microglial cells. Non-adherent cells with myelin debris were removed from these microglia-enriched cultures and transferred into poly-L-lysine coated 10 cm tissue culture plates in order to achieve adherence of astrocytes. Plates were incubated for 48 hr, after which the culture medium containing myelin debris and non-adherent cells was removed and used to prepare oligodendroglial cell cultures as described below. Both microglial- and astrocyte-enriched cultures were grown for 6 to 7 days before their mRNAs were extracted. Immunostaining with antibodies against CD68 (Dako, Mississauga, ON, Canada) which stains microglia as well as macrophages, and glial fibrillary acidic protein (GFAP, Dako), which is a marker of astrocytes, showed that the microglia-enriched cultures contained 93.5 ± 3.6 % (N = 4) microglial cells, while astrocyte-enriched cultures contained 85.7 ± 3.4 % (N = 4) astrocytes.

### Cell culture: oligodendroglial cells

These were prepared as described before [2]. Briefly, cell culture media containing myelin debris and non-adherent cells that were removed from astrocyte-enriched cultures were used to extract oligodendroglial cells. The non-adherent cells were collected by centrifugation at 275 × g for 10 min and replated onto uncoated 10 cm tissue culture plates for another 24 hr. Subsequently, the cell culture medium containing floating cells was transferred to 50 ml tubes and Lymphoprep solution (Axis-Shield, Oslo, Norway) used to reduce the amount of contaminating myelin debris. For this purpose, 10 ml of Lymphoprep solution was carefully placed under the oligodendrocyte cell suspension and the density gradient was centrifuged at 325 × g for 10 min. The interphase was collected and transferred to a 50 ml centrifuge tube. Fresh culture medium was added and the suspension was centrifuged at 275 × g for 7 min. The cell pellet was resuspended and the oligodendrocyte cultures seeded onto 60 mm plastic culture dishes. Immunostaining with anti-O4 antibody (Chemicon International, Temecula, CA), which is a marker of oligodendrocytes, showed that the oligodendrocytes-enriched cultures contained 95.3 ± 4.4 % (N = 4) oligodendrocytes.

### RNA isolation and cDNA synthesis by reverse transcription

Total RNA from oligodendroglial cells, microglia, and astrocytes were isolated by the acid guanidium thiocyanate-phenol-chloroform method. Two μg of the RNA was then used to prepare cDNA. RNA was treated with 10 U of DNase I (Gibco BRL, Life Technologies) for 60 min at 37°C in 25 μl of 1 × reverse transcriptase buffer (50 mM Tris-HCl, 75 mM KCl, 3 mM MgCl_2_) containing 40 U of RNase inhibitor (Pharmacia Biotech) and 1 mM dithiothreitol (DTT), following by incubation at 85°C for 5 min to inactivate the enzyme. Reverse transcription was performed at 42°C for 90 min in 50 μl of the following mixture: 1 × reverse transcriptase buffer containing 2 μg of RNA, 5 mM DTT, 0.2 μg random hexamer primers (Pharmacia Biotech), 1 mM deoxynucleotides (Gibco BRL, Life Technologies), 40 units of RNase inhibitor, and 400 units of SuperScript II reverse transcriptase (Invitrogen Life Technologies, Burlington, ON, Canada). At the end of the incubation period, the enzyme was inactivated by heating at 65°C for 10 min [29].

### Polymerase chain reaction

PCR amplification was carried out in a 25 μl reaction mixture containing 1 × GeneAmp PCR buffer II (Perkin Elmer, Foster City, CA), 1.25 units AmpliTaq Gold DNA polymerase (Perkin Elmer), 2 mM MgCl_2 _(Perkin Elmer), 200 μM dNTPs (Gibco BRL, Life Technologies) and 0.5 μM of each specific primer (Table 1). The mixture was prepared before the addition of 1.25 μl of cDNA. PCR amplification was carried out using an MJResearch (Boston, MA) programmable thermal controller. The amplification program consisted of an initial denaturation step at 94°C, which was extended to 9 min in order to activate AmpliTaq Gold enzyme. The remaining cycles were 1 min at 94°C, 1 min at 55°C and 1 min at 72°C. The number of cycles performed was 27 for glyceraldehyde-3-phosphate dehydrogenase (G3PDH), 30 for CD59, C1-inh and MCP, and 32 for DAF. After amplification, PCR products were separated on a 6% polyacrylamide gel and visualized by incubation for 10 min in a solution containing 10 ng/ml of ethidium bromide. Polaroid photographs of the gels were taken.

**Table 1:** Oligonucleotide primers used for PCR, and the corresponding restriction endonucleases used for product confirmation.

Gene	Sequence (5' → 3')	Fragment size (introns)	Genbank accession No	Restriction enzymes used and the expected sizes of digestion products (bp)
C1 inh-F	GTT GGG GGA TGC TTT GGT AGA TTT C	332	M13690	Sau 3AI (246, 86)
C1 inh-R	TTA GGA CTC TGG GGC TGC TGC TGT A	(2 introns)		
CD59-F	CTG CTG CTC GTC CTG GCT GTC TTC T	280	M34671	Pst I (233, 47)
CD59-R	TCC CAC CAT TTT CAA GCT GTT CGT T	(2 introns)		
MCP-F	CAA TTC AGT GTG GAG TCG TGC TGC	265	Y00651	Sau 3AI (193, 72)
MCP-R	TGA GGC ACT GGA CGC TGG AGA T	(unknown)		
DAF-F	GTA CTG TGA ATA ATG ATG AAG GAG	364	M30142	Hae III (330, 34)
DAF-R	TCT TAA CTC TTC TTT GGC TAA GTC	(unknown)		
G3PDH-F	CCA TGT TCG TCA TGG GTG TGA ACC A	251	X01677	Dde I (168, 83)
G3PDH-R	GCC AGT AGA GGC AGG GAT GAT GTT C	(2 introns)		

### PCR primer design and restriction analyses

Primers were designed to span introns so that cDNA-derived PCR products would be of different sizes to those produced if genomic DNA was amplified (see Table 1). DAF and MCP were exceptions, since only cDNA sequences were available. Primers were synthesized either by Sigma-Aldrich or ID Labs (London, ON, Canada). The primer sequences and predicted PCR fragment sizes are listed in Table 1, along with the names of the enzymes used for restriction digest analysis of each PCR fragment. The restriction digestion reactions were carried out at 37°C for 2 hr in the presence of 1 × the appropriate buffer provided by the suppliers (Invitrogen, Life Technologies and New England Biolabs, Mississauga, ON, Canada). The digested PCR products were analyzed on a 6% polyacrylamide gel (data not shown). In all cases the restriction fragments observed were of the predicted size (see Table 1).

### Statistical analysis

The data are presented as means ± s.e.m. The significance of difference between values was estimated by means of one-way analysis of variance (ANOVA) with Fisher's LSD post-hoc test. P < 0.05 was considered to show statistically significant differences.

### Double fluorescence immunocytochemical analysis

Oligodendrocytes, astrocytes, and microglia were harvested and air-dried on glass slides. Cells were then fixed with 4% paraformaldehyde for 10 min and permeabilized with 0.2% Triton X-100 in phosphate-buffered saline (PBS) for 5 min. For inactivation of endogenous peroxidase, cells were incubated with 0.3% H_2_O_2 _for 30 min. Blocking was performed for 1 hr at room temperature in 5% skim milk.

For double fluorescence immunostaining, cells were incubated at room temperature overnight with a primary antibody in 1% normal serum. The primary antibody and the dilution used in the first cycle were as follows: O4 (Chemicon International, 1: 100) for oligodendrocytes, GFAP (Dako, 1: 10,000) for astrocytes, CD68 (DAKO, 1: 50) for microglia. Cells were then treated for 2 hr at room temperature with a biotin conjugated anti-mouse IgM (Vector Laboratories, Burlingame, CA, 1: 200) secondary antibody for O4, a biotin conjugated anti-rabbit IgG (Vector Laboratories, 1: 200) secondary antibody for GFAP and a biotin conjugated anti-mouse IgG (Vector Laboratories, 1: 200) secondary antibody for CD68. Then cells were incubated with Texas Red Avidin DCS (Vector Laboratories) for 1 hr. The primary antibody and the dilution used in the second cycle were as follows: for C1-inh, goat anti-C1-inhbitor (Quidel, San Diego, CA, 1: 50); for CD59, mouse anti-CD59 (Serotec Ltd, Oxford, UK, 1: 10) or rat anti-CD59 (Serotec, 1: 25). Cells were incubated at 4°C for 3 days with a primary antibody in 1% serum corresponding to the secondary antibody type. Cells were then treated for 2 hr at room temperature with FITC-conjugated anti-mouse IgG (Vector Laboratories, 1: 200), anti-goat IgG (Santa Cruz Biotechnology, Santa Cruz, CA, 1: 200), or anti-rat IgG (Cappel, Durham, NC, 1: 200). The glass slides were then rinsed with distilled water, and a drop of Vectashield mounting medium (Vector Laboratories) placed on the slide.

## Results

### RT-PCR

**Figure 1:**
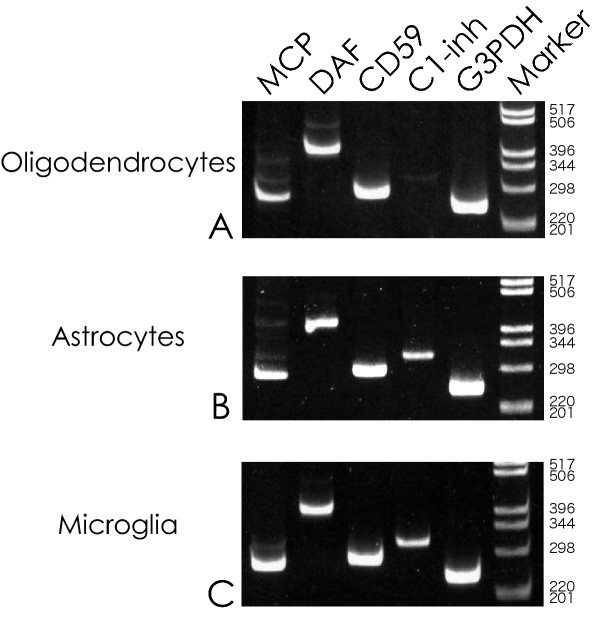
Demonstration of RT-PCR products. Polaroid photographs of typical ethidium bromide-stained gels of RT-PCR products from oligodendrocytic (Fig. 1A), astrocytic (Fig. 1B) and microglial (Fig. 1C) RNA extracts. Lanes for individual mRNA products are indicated in the legend at the top. Size markers are in the right lanes. MCP, membrane cofactor protein (265 bp); DAF, decay-accelerating factor (364 bp); CD59 (280 bp); C1-inh, C1-esterase inhibitor (332 bp); G3PDH, glyceraldehyde-3-phosphate dehydrogenase (251 bp).

RT-PCR was carried out using primers for C1-inh, CD59, DAF and MCP. The housekeeping gene G3PDH was amplified in parallel with each RT-PCR run as an internal standard. Figure 1 illustrates the bands obtained for each of the RT-PCR products from oligodendrocytes (Fig. 1A), astrocytes (Fig. 1B) and microglia (Fig. 1C). Specificity of each of the products was established by endonuclease digestion (Table 1).

### Semi-quantitative RT-PCR analysis

**Figure 2:**
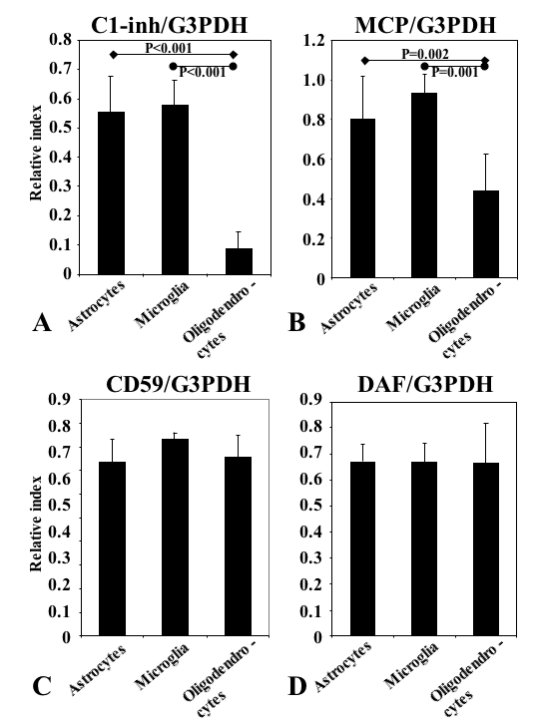
A comparison of relative complement inhibitor expression level between oligodendrocytes, astrocytes and microglia. The data were estimated by one-way analysis of variance (ANOVA) with Fisher's LSD post-hoc test (A and B; P < 0.05 was considered to show statistically significant differences).

To compare the ratio of each of the complement inhibitors to G3PDH, statistical analysis was performed by means of one-way ANOVA with Fisher's LSD post-hoc test (Fig. 2). The overall mean ± s.e.m. for C1-inh/G3PDH was 0.55 ± 0.12 (N = 5) in astrocytes, 0.58 ± 0.09 (N = 3) in microglia and 0.09 ± 0.06 (N = 12) in oligodendrocytes (Fig. 2A). Oligodendrocytes showed a highly significant difference from astrocytes and microglia (Fig. 2A; P < 0.001 by one-way ANOVA with Fisher's LSD post-hoc test). For MCP/G3PDH, the ratios were 0.80 ± 0.22 (N = 5) in astrocytes, 0.93 ± 0.10 (N = 3) in microglia and 0.44 ± 0.19 (N = 12) in oligodendrocytes. Oligodendrocytes showed a significant difference from astrocytes and microglia (Fig. 2B; P = 0.002 vs. astrocytes and P = 0.001 vs. microglia by one-way ANOVA with Fisher's LSD post-hoc test). The corresponding means for CD59/G3PDH were 0.73 ± 0.10 (N = 5) in astrocytes, 0.83 ± 0.03 (N = 3) in microglia and 0.76 ± 0.09 (N = 14) in oligodendrocytes (Fig. 2C). The corresponding means for DAF/G3PDH were 0.67 ± 0.07 (N = 5) in astrocytes, 0.67 ± 0.07 (N = 3) in microglia and 0.66 ± 0.15 (N = 14) in oligodendrocytes (Fig. 2D). There were no significant differences between the three cell types for CD59 and DAF. Each N represents a different patient.

### Double fluorescence immunohistochemistry

**Figure 3:**
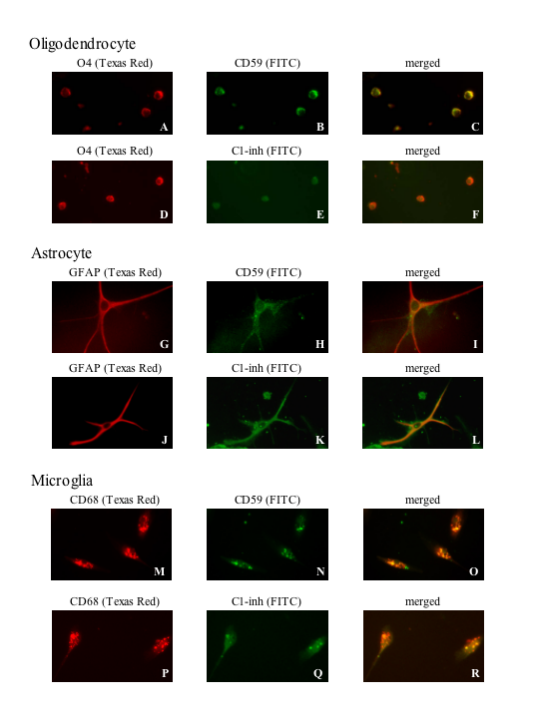
Double fluorescence immunohistochemistry of oligodendrocytes, astrocytes and microglia. Double fluorescence immunostaining for O4 and CD59 or C1-inh is demonstrated in A-F. In A and D, cells of typical oligodendroglial morphology were stained in the initial cycle for the specific oligodendroglial marker O4. Detection is by a Texas Red-conjugated secondary antibody. Second cycle staining for CD59 (B) and C1-inh (E) are shown. The detections are by an FITC-linked green fluorescent secondary antibody. In C and F, double immunofluorescences are shown in which the cells appear yellow, demonstrating colocalization of O4 with CD59 or C1-inh. Double fluorescence immunostaining of astrocytes for GFAP and CD59 or C1-inh is demonstrated in G-L. In G and J, cells of typical astrocytic morphology are stained in the initial cycle for the specific astroglial marker GFAP. Detection is by a Texas Red-conjugated secondary antibody. Second cycle staining for CD59 (H) and C1-inh (K) is shown with an FITC-linked green fluorescent secondary antibody. In I and L, double immunofluorescences are shown in which the cells appear yellow, demonstrating colocalization of GFAP with CD59 or C1-inh. Double fluorescence immunostaining for microglia using the specific marker CD68 and CD59 or C1-inh is demonstrated in M-R. In M and P, cells of typical microglial morphology are stained by CD68 with detection by a Texas Red-conjugated secondary antibody. Second cycle staining for CD59 (N) and C1-inh (Q) are shown. The detections are by an FITC-linked green fluorescent secondary antibody. In O and R, double immunofluorescences are shown in which the cells appear yellow, demonstrating colocalization of CD68 with CD59 or C1-inh. (Magnification: × 200)

In order to establish identity between oligodendroglial cells, astrocytes or microglia and cells expressing the complement inhibitor proteins CD59 or C1-inh, double fluorescence immunostaining was carried out. Oligodendrocytes were detected by O4 staining with a Texas Red tagged secondary antibody (Fig. 3A and 3D) in the first cycle and CD59 (Fig 3B) or C1-inh staining (Fig. 3E) detected with a green FITC tagged antibody in the second cycle. Astrocytes were detected by GFAP staining with a Texas Red tagged secondary antibody (Fig. 3G and 3J) in the first cycle and CD59 staining (Fig 3H) or C1-inh staining (Fig. 3K) detected with a green FITC tagged antibody in the second cycle. Microglia were detected by CD68 staining with a Texas Red tagged secondary antibody (Fig. 3M and 3P) in the first cycle, and CD59 staining (Fig 3N) or C1-inh staining (Fig. 3Q) detected with a green FITC tagged antibody in the second cycle. With double fluorescent excitation, all cells fluoresced yellow (Fig. 3C,3F,3I,3L,3O,3R), indicating colocalization of O4 with CD59 or C1-inh, GFAP with CD59 or C1-inh, and CD68 with CD59 or C1-inh.

## Discussion

This report shows that human oligodendrocytes express a much lower level of mRNA for C1-inh than astrocytes and microglia, and a significantly lower level of mRNA for MCP. The mRNA levels of CD59 and DAF were comparable in all the three cell types. Overall our data suggest that oligodendroglial cells, in common with other cell types, can produce complement inhibitors, but at a significantly lower level for C1-inh and MCP.

It has already been reported that human neurons and Schwann cells [24], neuroblastoma cell lines [18], astrocytes [23], astrocytoma cell lines [25,26], the HOG human oligodendroglial cell line [1] and oligodendrocytes [21,22] produce some or all of the complement inhibitor proteins and their mRNAs.

Activation of the complement cascade and deposition of activated complement fragments occur in non-infectious diseases such as multiple sclerosis, Pick's disease, Alzheimer's disease and other neurodegenerative conditions [15,16,30-34]. Complement inhibitors may play an important role in preventing such pathology.

Full activation of the complement cascade requires overcoming a series of endogenous inhibitory factors. Oligodendrocytes are vulnerable to complement attack, which is particularly observed in multiple sclerosis [35-37] and this vulnerability may be related to a deficiency of their ability to express complement regulatory proteins [22], particularly C1-inh.

Sporadic complement attack, in the form of complement activated oligodendroglia (CAO) is also observed in a number of neurodegenerative conditions [38,39], including Alzheimer's, Pick's, Huntington's and Parkinson's diseases, amyotrophic lateral sclerosis, progressive supranuclear palsy, Shy-Drager syndrome, argyrophilic grain dementia and pallido-nigral luysial atrophy [38,39]. The source of the complement proteins that become activated is unknown, but the data presented here suggest that oligodendrocytes are vulnerable to complement attack because of a low expression of C1-inh and MCP.

## Conclusions

These results suggest that the lower expression of C1-inh and MCP by oligodendrocytes could contribute to their vulnerability in several neurodegenerative and inflammatory diseases of the central nervous system, particularly multiple sclerosis.

## List of abbreviations

analysis of variance (ANOVA)

central nervous system (CNS)

complement activated oligodendroglia (CAO)

complement receptor 1 (CR1)

decay-accelerating factor (DAF)

dithiothreitol (DTT)

fluorescein isothiocyanate isomer (FITC)

glyceraldehyde-3-phosphate dehydrogenase (G3PDH)

glial fibrillary acidic protein (GFAP)

homologous restriction factor (HRF)

membrane cofactor protein (MCP)

phosphate-buffered saline (PBS)

## Competing interests

None declared.

## Authors' contributions

MH was responsible for the majority of the experimental studies, and for writing the manuscript. AK contributed to the cell culture and the editing of the manuscript. PLM contributed to the conception, interpretation of results and the writing and editing of the manuscript.
